# Comparison of short-term outcomes between transthoracic and robot-assisted transmediastinal radical surgery for esophageal cancer: a prospective study

**DOI:** 10.1186/s12885-021-08075-1

**Published:** 2021-03-31

**Authors:** Shuntaro Yoshimura, Kazuhiko Mori, Motonari Ri, Susumu Aikou, Koichi Yagi, Yukinori Yamagata, Masato Nishida, Hiroharu Yamashita, Sachiyo Nomura, Yasuyuki Seto

**Affiliations:** 1 Department of Gastrointestinal Surgery, Graduate School of Medicine, The University of Tokyo Hospital, 7-3-1 Hongo, Bunkyo-ku, Tokyo, 113-8655 Japan; 2 Department of Gastrointestinal Surgery, Mitsui Memorial Hospital, 1 Kanda Izumi, Chiyoda-ku, Tokyo, 101-8643 Japan; 3 Department of Gastric Surgery, National Cancer Center Hospital, 5-1-1 Tsukiji, Chuo-ku, Tokyo, 104-0045 Japan

**Keywords:** Esophageal cancer, Esophagectomy, Minimally invasive esophagectomy, Robot-assisted esophagectomy

## Abstract

**Background:**

The present study aimed to assess the lower invasiveness of robot-assisted transmediastinal radical esophagectomy by prospectively comparing this procedure with transthoracic esophagectomy in terms of perioperative outcomes, serum cytokine levels, and respiratory function after surgery for esophageal cancer.

**Methods:**

Patients who underwent a robot-assisted transmediastinal esophagectomy or transthoracic esophagectomy between April 2015 and March 2017 were included. The perioperative outcomes, preoperative and postoperative serum IL-6, IL-8, and IL-10 levels, and respiratory function measured preoperatively and at 6 months postoperatively were compared in patients with a robot-assisted transmediastinal esophagectomy and those with a transthoracic esophagectomy.

**Results:**

Sixty patients with esophageal cancer were enrolled. The transmediastinal esophagectomy group had a significantly lower incidence of postoperative pneumonia (*p* = 0.002) and a significantly shorter postoperative hospital stay (*p* < 0.0002). The serum IL-6 levels on postoperative days 1, 3, 5, and 7 were significantly lower in the transmediastinal esophagectomy group (*p* = 0.005, 0.0007, 0.022, 0.020, respectively). In the latter group, the serum IL-8 level was significantly lower immediately after surgery and on postoperative day 1 (*p* = 0.003, 0.001, respectively) while the serum IL-10 level was significantly lower immediately after surgery (*p* = 0.041). The reduction in vital capacity, percent vital capacity, forced vital capacity, and forced expiratory volume at 1.0 s 6 months after surgery was significantly greater in the transthoracic esophagectomy group (*p* < 0.0001 for all four measurements).

**Conclusions:**

Although further, large-scale studies are needed to confirm our findings, robot-assisted transmediastinal esophagectomy may confer short-term benefits in radical surgery for esophageal cancer.

**Trial registration:**

This trial was registered in the UMIN Clinical Trial Registry (UMIN000017565 14/05/2015).

## Background

Successful treatment options for esophageal cancer include chemotherapy and radiotherapy, but surgical treatment also plays an important role [1]. However, a radical esophagectomy entails postoperative complications and can result in postoperative mortality. According to the National Clinical Database (a large-scale database in Japan), the rate of postoperative complications following an esophagectomy is approximately 43%, and the postoperative mortality rate is approximately 3% [2]. Video-assisted thoracoscopic esophagectomy is regarded as a minimally invasive surgical technique for esophageal cancer [3–5] and has the advantage of less postoperative pain and faster lung capacity recovery than seen in transthoracic esophagectomy (TTE) [6, 7]. However, a study comparing TTE with video-assisted thoracoscopic esophagectomy based on the aforementioned National Clinical Database showed no significant decrease in the length of hospital stay or any significant reduction in postoperative mortality. Furthermore, no significant reduction in the postoperative pneumonia rate was observed (15.5% for TTE and 15.0% for video-assisted thoracoscopic esophagectomy). Additionally, TTE reportedly increased postoperative complications requiring reoperation [2]. Despite being considered a minimally invasive procedure, video-assisted thoracoscopic esophagectomy had no significant positive impact on the pulmonary complication rate. Transhiatal esophagectomy is also a favored choice and is associated with lower perioperative morbidity, but the oncological outcome of this approach is generally considered to be inferior because fewer lymph nodes can be harvested than in the transthoracic approach [8].

We developed transmediastinal esophagectomy (TME), a nontransthoracic esophagectomy with radical mediastinal lymphadenectomy combining a robotic transhiatal approach with the video-assisted cervical approach reported previously by the authors [9]. In our previous retrospective study, we confirmed the practicability and safety of this procedure, demonstrated the equivalence of its oncological outcome with that of TTE in terms of the number of harvested lymph nodes and surgical margin pathology, and assessed the reduction in respiratory complications [10]. In the present, retrospective study, postoperative pneumonia was not observed in the TME group but was observed in 15% patients in the TTE group, and the length of postoperative hospital stay also significantly decreased.

The present study aimed to assess the lower invasiveness and the utility of the robot-assisted transmediastinal radical esophagectomy by prospectively evaluating the postoperative outcomes, changes in serum cytokine levels, and respiratory function over time.

## Methods

The study patients were recruited at the Department of Gastrointestinal Surgery of the University of Tokyo Hospital between April 2015 and March 2017. The inclusion criteria were: (1) histologically proven esophageal cancer; (2) a T0–3 N0–2 M0 stage tumor according to the TNM Classification of Malignant Tumors, 7th edition; (3) age 20 years or older to 85 years or younger; (4) European Clinical Oncology Group Performance Status (ECOG-PS) ≦1; (5) good enough general health to tolerate a conventional open esophagectomy; (6) no concomitant malignancies; and (7) no preoperative radiotherapy. Patients with a history of surgery for other malignancies were excluded.

All the patients were offered the option of surgery (TTE or robot-assisted TME), and robot-assisted TME was performed in patients who elected to undergo this procedure despite lack of coverage under National Health Insurance. TTE was performed for all the remaining patients. The perioperative outcomes, serum cytokine levels, and respiratory function between the TME and TTE groups were compared.

To measure the serum cytokine levels, blood samples were collected preoperatively, immediately after surgery, and on postoperative days 1, 3, 5, and 7. The samples were immediately stored at 4 °C and centrifuged for 10 min at 3000 rpm, and the supernatant was cryopreserved at − 80 °C. After thawing, serum IL-6, IL-8, and IL-10 levels were measured using a BD CBA Human Inflammatory Cytokines Kit (BD Biosciences Inc., NJ, USA). All the samples were analyzed using BD Accuri C6 (BD Biosciences Inc., NJ, USA).

For respiratory function assessment, vital capacity (VC), forced vital capacity (FVC), and forced expiratory volume at 1.0 s (FEV 1.0) were measured preoperatively and at postoperative six months. The two groups were then compared in terms of pre- and postoperative changes.

The present, prospective study was approved by The University of Tokyo’s institutional review board. All 60 study participants provided written informed consent. This trial was registered in the UMIN Clinical Trial Registry (UMIN000017565 14/05/2015).

### Surgical methods

Details of the procedure of TME and TTE have been described previously [9, 11]. In brief, TME was performed in the supine position. LN dissection in the cervical and the abdominal fields was performed simultaneously via the mediastinoscopic and laparoscopic approach. The robotic surgical device, da Vinci S (Intuitive Surgical, Sunnyvale, CA, USA), was used to perform the transhiatal procedure through the abdominal ports. In the dissections in the cervical procedure via the collar incision and the da Vinci procedure via the transhiatal approach, the whole esophagus and the dissected mediastinal LNs were able to be freed from adhesion and attachments.

The TTE group received a right anterolateral thoracotomy via the fourth intercostal space with a two or three-field lymphadenectomy and intrathoracic anastomosis. After the creation of gastric conduit, the posterior mediastinal route was used, and anastomosis was performed using a 25-mm, circular stapler. All surgical procedures were performed by one, experienced surgeon (Y.S).

### Statistical analysis

All statistical analyses were performed using JMP 11.0 (SAS Institute Inc. NC, USA). Wilcoxon’s rank-sum test was used in the continuity scale test for analysis of the patient background of each group and perioperative data, and Fisher’s exact test was used to assess proportional differences in the nominal scale. To identify risk factors for the onset of pneumonia following surgery for esophageal cancer, univariate analysis was conducted using the factors listed below as independent variables, and multivariate analysis was conducted with logistic regression. The independent variables were sex, age (< 75 years / ≥75 years), smoking habit (smoker / non-smoker), surgical procedure (TTE / TME), operative duration (mean < 434 min / ≥434 min), estimated blood loss (mean < 410 ml / ≥410 ml), pathological staging (stage I–II / stage II–IV), and preoperative respiratory abnormality (with / without). The presence of a preoperative respiratory abnormality was determined by observation of either restrictive or obstructive ventilator impairment. Serum inflammatory cytokine levels and the respiratory function were compared between the groups using Student’s t-test. The respiratory function was compared preoperatively and at 6 months postoperatively using a paired Student’s t-test for each surgical procedure. *P* < 0.05 was considered to indicate statistical significance.

## Results

Seventy-eight patients with esophageal cancer between April 2015 and March 2017 who met the inclusion criteria were found. Among these patients, 18 had a history of surgery for another malignancy. In total, 60 patients were enrolled, and all were included in our analyses. Table 1 shows the background of 25 patients who underwent TME and 35 patients who underwent TTE. Cervical anastomosis was significantly more common in the TME group than in the TTE group (*p* < 0.0001). R0 resection (no residual tumor) was performed in 25 patients (100%) in the TME group and 34 patients (97.1%) in the TTE group. One patient (2.9%) in the TTE group underwent R1 resection, i.e., resection for a microscopic residual tumor. There was no case of R2 resection, i.e., resection for a macroscopic residual tumor, in either the TME or TTE group. Although there was no significant difference in the pathological stages between the groups, more patients in the TTE group had an advanced clinical and pathological tumor stage (cT and pT) (*p* = 0.0043, 0.01).

**Table 1:** Clinicopathological characteristics

	TME (*n* = 25)	TTE (*n* = 35)	*p* value*
Median age (range)	66 (43–78)	66 (51–82)	0.96
Sex (M/F)	23/2	28/7	0.28
Median BMI (range)	22.4 (17.9–28.6)	22.2 (17.5–29.7)	0.55
ECOG-PS (0/1)	9/16	10/25	0.58
Brinkman Index (0/ 1–600/ 601–1200/ 1201-)	7/10/5/3	7/12/13/3	0.55
Location (Proximal/Middle/Distal/EGJ)	2/18/4/1	2/19/8/6	0.35
Anastomosis site (Cervical/Intrathoracic)	25/0	7/28	<.0001*
Number of three-field lymphadenectomies (%)	22 (88.0%)	23 (65.7%)	0.07
Clinical classification			
cT Status (0/1/2/3)	0/17/8/0	2/13/10/10	0.0043*
cN Status (0/1/2)	20/5/0	28/6/1	1.00
Pathological classification			
pT Status (0/1/2/3)	0/19/3/3	1/16/2/16	0.01*
pN Status (0/1/2/3)	13/6/5/1	16/12/5/2	0.16
pStage (0/IA/IB/IIA/IIB/IIIA/IIIB/IIIC)	0/10/2/1/5/6/0/1	1/11/1/3/5/8/5/1	0.52
Resection status (R0/R1/R2)	25/0/0	34/1/0	1.00
Histological type (SCC/AC/Other)	24/0/1	31/3/1	0.38
	Number of cases (%)		*p* value
Neoadjuvant chemotherapy: n (%)	2 (8.0%)	11 (31.4%)	0.054
Adjuvant chemotherapy: n (%)	9 (36.0%)	11 (31.4%)	0.78

*Fisher’s exact test. AC, adenocarcinoma; BMI, body mass index; EGJ, esophagogastric junction; SCC, squamous cell carcinoma; TME, transmediastinal esophagectomy; TTE, transthoracic esophagectomy

### Perioperative outcomes

Table 2 shows the perioperative outcomes, incidence of postoperative complications with a grade higher than 2 in the Clavien-Dindo Classification [12], and incidence of postoperative pneumonia. The diagnosis of postoperative pneumonia was made in accordance with the Japanese Respiratory Society’s Guidelines for Hospital Acquired Pneumonia in Adults [12]. The TME group had a significantly longer operative time (*p* < 0.0001) but significantly less blood loss (*p* = 0.0004) than the TTE group. In terms of postoperative complications, the TME group had no cases of postoperative pneumonia and significantly fewer cases of pneumonia in general (0% vs. 31.4%; *p* = 0.002). The incidence of anastomotic leakage was higher (though not significantly so) (32% vs. 11%; *p* = 0.099), and the median postoperative hospital stay was significantly shorter (18 days vs.25 days; *p* = 0.0002), in the TME group.

**Table 2:** Postoperative outcomes

	TME (n = 25)	TTE (n = 35)	
	Median (range)		*p* value*
Duration of operation (min)	508 (417–612)	377 (274–469)	<.0001*
Blood loss (ml)	215 (20–985)	435 (80–1380)	.0004*
Numbers of lymph node yield	67 (19–92)	55 (32–102)	0.12
Hospital stay (days)	18 (11–35)	25 (16–99)	.0002*
	Number of caces (%)		*p* value**
Pneumonia	0 (0%)	11 (31.4%)	0.0016**
Reintubation	2 (8%)	5 (14.3%)	0.69
Anastomotic leak	8 (32%)	4 (11.4%)	0.099
Recurrent laryngeal nerve palsy	2 (8%)	4 (11.4%)	1.00
Chylothrax	1 (4%)	2 (5.7%)	1.00
Surgical stie infection	1 (4%)	1 (2.9%)	1.00
In-hospital mortality	0 (0%)	0 (0%)	1.00

*Wilcoxon rank sum test. **Fisher’s exact test. *TME* Transmediastinal esophagectomy, *TTE* Transthoracic esophagectomy

Table 3 shows the assessment of the risk factors for postoperative pneumonia. Univariate analysis revealed that TTE (*p* = 0.0002) and age (*p* = 0.0051) were significant risk factors of postoperative pneumonia. This finding was corroborated by multivariate analysis, which also revealed that TTE (*p* = 0.0006) and age (*p* = 0.014) were risk factors of postoperative pneumonia.

**Table 3:** Multivariate analysis of variables predicting postoperative pneumonia

Univariate				Multivariate		
Variables	OR	95% CI	*p* value	OR	95% CI	*p* value
Sex:Male	1.95	0.22–17.45	0.52			
Smoking history: Yes	0.77	0.17–3.41	0.74			
Age ≧ 75	9.38	1.96–44.91	0.0051	9.17	1.57–76.65	0.014
Surgical approach: TTE	–	–	0.0002	–	–	0.0006
Operation duration ≧ 434 min	0.51	0.13–1.95	0.31			
Blood loss ≧ 410 ml	2.26	0.60–8.49	0.23			
pStage ≧ III	0.65	0.15–2.75	0.55			
Preoperative respiratory abnormality: Yes	1.97	0.49–8.00	0.35			

*CI* Confidence interval, *OR* Odds ratio, *TTE* Transthoracic esophagectomy

### Serum cytokines

**Fig. 1:**
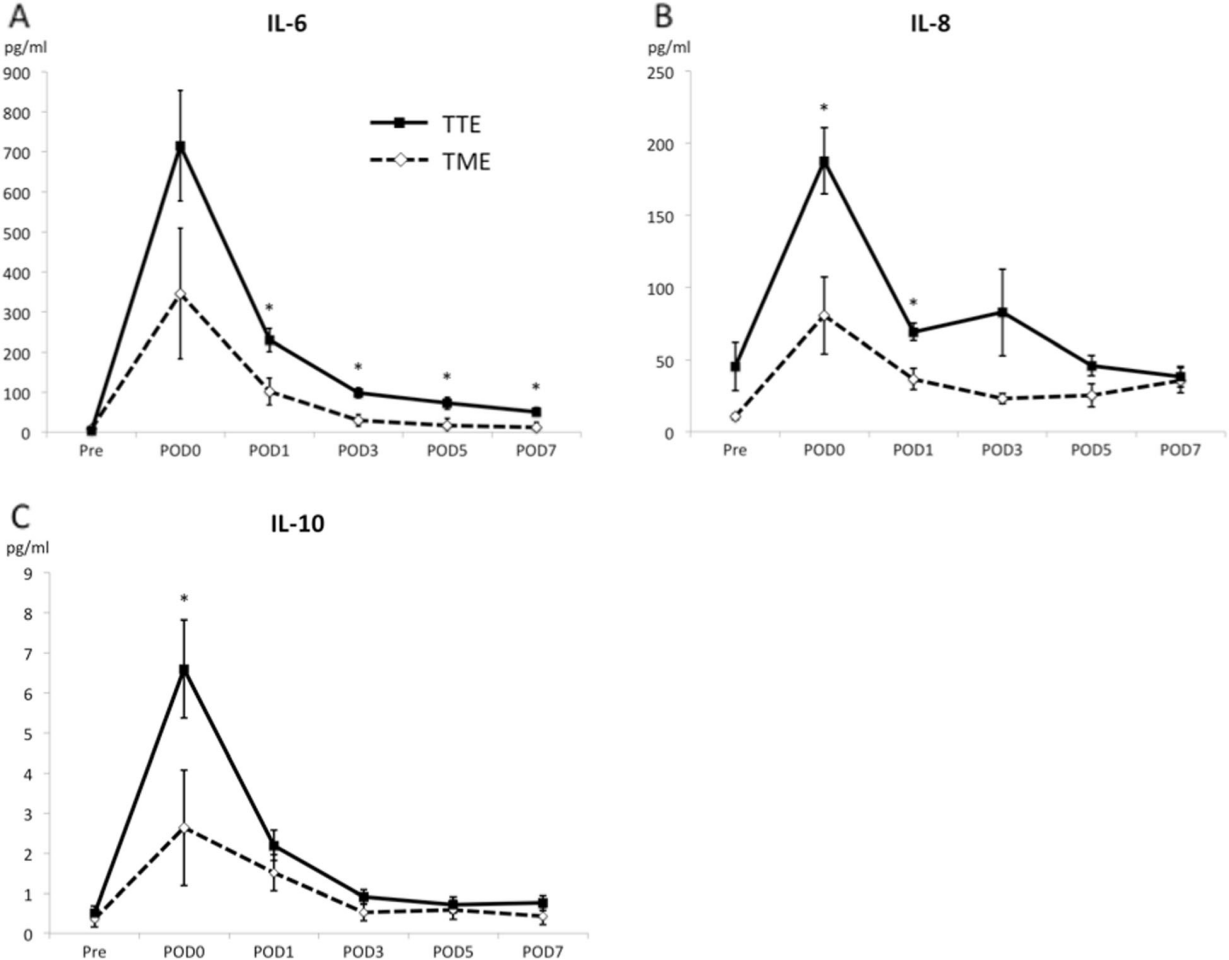
Comparison of preoperative and postoperative serum IL-6 (**a**), IL-8 (**b**), and IL-10 (**c**) levels in patients undergoing transthoracic esophagectomy or transmediastinal esophagectomy. Data are presented as the mean ± standard error of the mean

Figure 1a, b, and c show changes in serum IL-6, IL-8, and Il-10 levels in both groups preoperatively, immediately after surgery, and on postoperative days 1, 3, 5, and 7. The TME group showed a significantly lower IL-6 level on postoperative days 1, 3, 5, and 7 (*p* = 0.005, 0.0007, 0.022, 0.020, respectively), a significantly lower IL-8 level immediately after surgery and on postoperative day 1 (*p* = 0.003 and 0.001, respectively), and a significantly lower IL-10 level after surgery (*p* = 0.014).

### Respiratory function

**Fig. 2:**
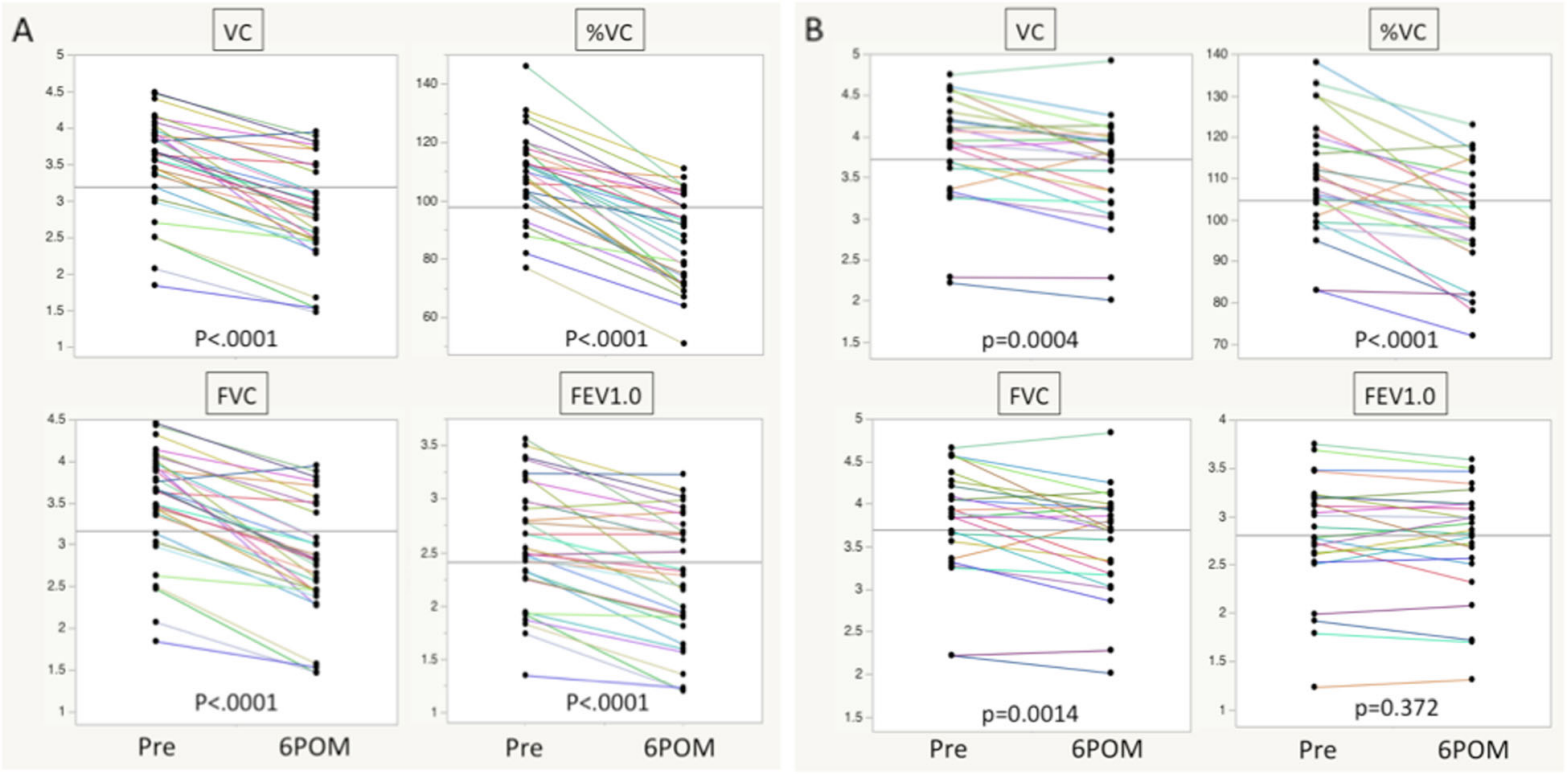
Comparison of VC, %VC, FVC, and FEV1.0 values measured preoperatively and at postoperative 6 months in the TTE (**a**) and TME (**b**) groups

In the TTE group, two patients died at postoperative 6 months due to esophageal cancer recurrence, and one patient did not undergo respiratory function testing due to a hospital transfer. Therefore, the respiratory function of 25 patients in the TME group and 32 patients in the TTE group was examined. Table 4 shows the mean, preoperative, respiratory function values of both groups, and Fig. 2a and b show changes in the respiratory function from before surgery to postoperative 6 months per group. No significant difference was observed between the groups in terms of preoperative VC, %VC, FVC or FEV1.0. In both groups, VC, %VC, and FVC were significantly lower at postoperative 6 months than before surgery (TME group: *p* = 0.0004, < 0.0001, and 0.0014, respectively; TTE group: *p* < 0.0001, < 0.0001, and < 0.0001, respectively). Furthermore, the postoperative FEV1.0 value decreased to a significantly greater degree in the TTE group (*p* < 0.0001) than in the TME group (*p* = 0.372).

**Table 4:** Preoperative respiratory function in TME and TTE

	TME (n = 25)	TTE (*n* = 32)	*p* value
VC (L)	3.85 ± 0.13	3.54 ± 0.12	0.081
%VC	110.0 ± 2.82	109.5 ± 2.49	0.9
FVC (L)	3.81 ± 0.13	3.52 ± 0.11	0.092
FEV1.0 (L)	2.82 ± 0.12	2.58 ± 0.10	0.12
FEV1.0%	75.7 ± 2.01	75.0 ± 1.78	0.81

Student t test.; *FEV1.0* Forced expiratory volume in 1 s, *FEV1.0%* Forced expiratory volume in 1 s percent predicted, *FVC* Forced vital capacity, *TME* Transmediastinal esophagectomy, *TTE* Transthoracic esophagectomy, *%VC* Vital capacity percent predicted, *VC* Vital capacity

**Fig. 3:**
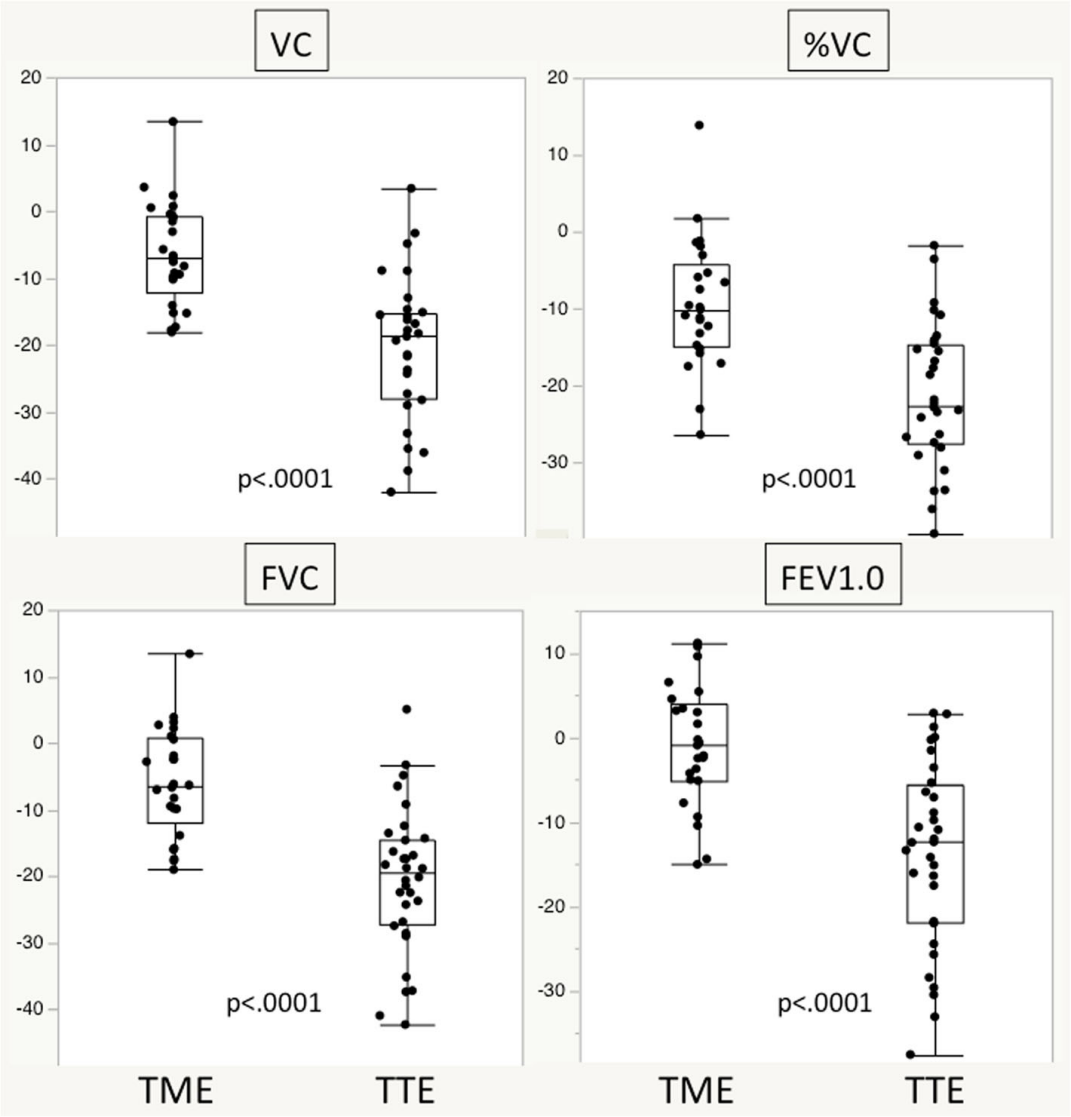
Comparison of reductions in VC, %VC, FVC, and FEV1.0 rate preoperatively and at postoperative 6 months in the TTE and TME groups

Figure 3 shows the mean rate of change for each respiratory parameter between postoperative 6 months and before surgery. VC, %VC, FVC, and FEV1.0 decreased by 20.2, 21.5, 20.5, and 13.7%, respectively, in the TTE group whereas the corresponding values decreased by 6.3, 9.4, 5.8, and 1.0%, respectively, in the TME group. The reduction rate in all the parameters was significantly larger in the TTE group (*p* < 0.0001 for all parameters).

## Discussion

The present, prospective study compared perioperative outcomes, postoperative serum cytokine levels, and postoperative respiratory function in patients in a TME and TTE group who underwent radical surgery for esophageal cancer. Although the operation time was longer, the incidence of postoperative pneumonia was significantly lower, and the length of postoperative hospital stay was shorter, in the TME group than in the TTE group, suggesting that robot-assisted transmediastinal radical esophagectomy can be effective as a minimally invasive surgery for esophageal cancer. Differences in surgical approach and postoperative respiratory function might have contributed to the reduction in postoperative pneumonia. TME preserves the respiratory muscles and avoids adhesion associated with thoracotomy; this likely explains the postoperatively preservation of the respiratory function in TME. Furthermore, the results of multivariate analysis showed that older age and TTE were risk factors of postoperative pneumonia.

The present study revealed that postoperative serum cytokine levels decreased to a significantly greater degree in the TME group than in the TTE group. In studies comparing postoperative serum cytokine levels in TTE and video-assisted thoracoscopic esophagectomy, patients undergoing the latter procedure had significantly lower postoperative IL-6, IL-8, and IL-10 level [13–16] and postoperative pneumonia incidence [15, 16]. The elevated serum IL-6 levels in TTE can reportedly be caused by exposure to oxygen in the lungs or by mechanical stimulation of the lungs [17–19], both of which are reduced in video-assisted thoracoscopic esophagectomy. These two factors may be altogether absent in TME.

In the present study, the lower invasiveness of TME was assessed using serum IL-6, IL-8, and IL-10 levels as markers to indicate the degree of surgical invasiveness. Despite the longer surgical time required for TME, this study found that elevation in the level of all the cytokines immediately after surgery was inhibited in patients who underwent this procedure.

Respiratory function was better preserved after TME than after TTE. Previous studies on respiratory function after esophageal cancer surgery demonstrated that the reduction in the rate of post-thoracotomy VC and FEV 1.0 was 26 and 16%, respectively, whereas the corresponding values following video-assisted thoracoscopic esophagectomy were 15 and 8%, respectively [7]. In the present study, VC and FEV 1.0 after TTE decreased by 20.2 and 13.7%, respectively, in line with the findings of previous studies [7]. However, after TME, the VC and FEV 1.0 reduction rate was 6.3 and 1.0%, respectively, suggesting that TME is more effective in preserving respiratory function than video-assisted thoracoscopic esophagectomy. Respiratory muscle dysfunction following a laparotomy and thoracotomy is caused by reduced contraction efficiency of the dissected muscles, postoperative pain, and inhibition of phrenic nerve activity due to stimulation of the internal organs [17]. Respiratory function is most reduced immediately after surgery and recovers gradually [20]. In addition, physiological stress to the ventilated and non-ventilated lungs by differential lung ventilation is thought to reduce respiratory function during a thoracotomy. However, such factors are often minimized or avoided in TME. The preservation of postoperative respiratory function observed in the present study may have contributed to the reduced postoperative pneumonia rate.

Complications following TME included a higher incidence of anastomotic leakage than seen with TTE. Anastomotic leakage following surgery for esophageal cancer is associated with cervical anastomosis [21, 22], which is likely to be caused by tension applied to the site of anastomosis, ischemia, venous return insufficiency, and/or pressure of the gastric tube at the thoracic inlet [22]. In TME, all patients undergo cervical anastomosis, and the aforementioned factors were considered to have caused the high anastomotic leakage incidence in the present study. However, the length of postoperative hospital stay was significantly shorter in the TME group, suggesting that anastomotic leakage in this group might not have adversely affected the length of hospital stay in the present study.

The present study included a large number of patients in the TTE group with an advanced tumor stage. Although there was no significant difference in the lymph node stage or pathological stage, it is possible that the comparison was not oncologically equal. Moreover, in the present study, the assignment of patients to the surgical procedure was not randomized, and differences in the patient background may have affected the effectiveness of TME.

## Conclusions

In conclusion, the present, prospective study indicated that TME might be a minimally invasive surgical procedure providing more, short-term benefits than TTE. However, additional studies should be conducted to evaluate the benefits of TME for patients with advanced esophageal cancer. Moreover, the present study did not compare TME with video-assisted thoracoscopic esophagectomy and included more confounding factors than a randomized trial.

## Data Availability

The datasets used and/or analyzed during the current study are available from the corresponding author on reasonable request.

## Abbreviations

ECOG-PS: European Clinical Oncology Group Performance Status
FEV 1.0: Forced expiratory volume at 1.0 s
FVC: Forced vital capacity
TME: Transmediastinal esophagectomy
TTE: Transthoracic esophagectomy
VC: Vital capacity
